# Experiences of membership in *munno mubulwadde (your friend indeed)* - a novel community-based health insurance scheme in Luwero district in rural central Uganda

**DOI:** 10.1186/s12913-023-10517-4

**Published:** 2024-01-17

**Authors:** Christine Nabanoba, Henry Zakumumpa

**Affiliations:** 1 Department of Social Work and Social Administration, Makerere University, Kampala, Uganda; 2 School of Public Health, Makerere University, Kampala, Uganda

**Keywords:** Universal health care, Health insurance, Catastrophic health expenditure, Health financing, Resource-limited settings

## Abstract

**Background:**

Community-Based Health Insurance (CBHI) schemes are recognized as an important health financing pathway to achieving universal health coverage (UHC). Although previous studies have documented CBHIs in low-income countries, the majority of these have been provider-based. Non-provider based schemes have received comparatively less empirical attention. We sought to describe a novel non-provider based CBHI *munno mubulwadde* (your friend indeed) comprising informal sector members in rural central Uganda to understand the structure of the scheme, the experiences of scheme members in terms of the perceived benefits and barriers to retention in the scheme.

**Methods:**

We report qualitative findings from a larger mixed-methods study. We conducted in-depth interviews with insured members (*n =* 18) and scheme administrators (*n =* 12). Four focus groups were conducted with insured members (38 participants). Data were inductively analyzed by thematic approach.

**Results:**

*Munno mubulwadde* is a union of ten CBHI schemes coordinated by one administrative structure. Members were predominantly low-income rural informal sector households who pay annual premiums ranging from $17 and $50 annually and received medical care at 13 scheme-contracted private health facilities in Luwero District in Central Uganda. Insured members reported that scheme membership protected them from catastrophic health expenditure during episodes of sickness among household members, and especially so among households with children under-five who were reported to fall sick frequently, the scheme enabled members to receive perceived better quality health care at private providers in the study district relative to the nearest public facilities. The identified barriers to retention in the scheme include inconvenient dates for premium payment that are misaligned with harvest periods for cash crops (e.g. maize corn) on which members depended for their agrarian livelihoods, long distances to insurance-contracted private providers, falling prices of cash crops which diminished real incomes and affordability of insurance premiums in successive years after initial enrolment.

**Conclusion:**

*Munno mubulwadde* was perceived by as a valuable financial cushion during episodes of illness by rural informal sector households. Policy interventions for promoting price stability of cash crops in central Uganda could enhance retention of members in this non-provider CBHI which is worthy of further research as an additional funding pathway for realizing UHC in Uganda and other low-income settings.

**Supplementary Information:**

The online version contains supplementary material available at 10.1186/s12913-023-10517-4.

## Background

Globally, there is growing consensus around the need to advance progress towards attainment of the universal health coverage (UHC) agenda [1]. UHC provides for extending social protection in health to the whole population in order to reduce financial barriers to health care access for the needy and to avoid catastrophic health expenditure [1, 2]. Among the salient pathways for attaining UHC is the route of social health insurance which is receiving increasing attention in global health agendas [1]. Social health insurance schemes generate additional resources in the chronically under-funded health systems in sub-Saharan Africa (SSA) [3].

Social health insurance is slowly gaining traction in SSA as a health care financing alternative to public finance taxation and out-of-pocket (OOP) expenditures [4]. Indeed, countries such as Rwanda which are implementing community-based health insurance are making vigorous efforts to extend coverage to the informal sector [4]. This is aimed at improving health outcomes and is increasingly attracting attention from governments and donors in SSA [4].

Almost 30% of the Ugandan population is classified as poor or live on five dollars (US$) or less a day [5]. According to the national health accounts in Uganda, more than a third of the poor who were ill did not seek care, compared with only 15% of higher income individuals [6]. Furthermore, 7.7% of poor households were faced with catastrophic health expenditure which is defined as exceeding 40% of disposable household income [6].

There has been considerable research on health insurance for the formal sector. However, there is less attention devoted to the informal sector in low–income countries where the majority of the population belong. Individuals working in the informal sector tend to avoid public facilities and instead opt for private health care or avoid health care altogether for those who can’t afford, incurring the risk of more extensive (and expensive) procedures down the line [7]. Hence, alternative means of financing health care financing in Uganda are a top priority on the public policy agenda [8]. Expanding access to Community-Based Health Insurance (CBHI) schemes for the informal sector and the poor is therefore an important policy option enshrined in the Uganda Health Sector Strategy [9, 10].

A recent WHO study in Uganda revealed that Ugandans spend 22% of their income on health care, and 6% of the poorest who have the highest number of health bills have to sell their household assets to meet costs on health care.

In March 2021, the Parliament of Uganda passed a national health insurance bill that outlines the general structure for a first-ever national social health insurance scheme in Uganda. It was passed with a pre-set benefits package that includes a range of essential health services including family planning services. The scheme was meant to be financed by a combination of employee and government contributions with the aim of providing health care cover for all Ugandans. However, the president of Uganda is yet to assent to this bill. The bill awaits re-consideration by the national legislature and is currently under the stewardship of the Uganda Ministry of Health. As such, Uganda continues to have some of the highest out-of-pocket costs for health in the region estimated at around 38% [11].

Community-Based Health Insurance (CBHI) is one of the mechanisms envisaged in the Uganda health sector strategic plan 2015/16-2019-2020 as one of the pathways to financing health services [9]. CBHI schemes are being advanced as one of the viable options for addressing health financing constraints in low and middle-incomes [12–14].

In Uganda, the CBHI schemes which are relatively well documented are those which are provider-based [15, 16]. On the other hand, there is comparatively less empirical attention towards CBHI schemes initiated and managed by community members themselves in Uganda which account for 14% of all CBHI schemes in Uganda [17, 18]. This paper begins to fill this gap. This paper focuses on a community-led health insurance schemes in Luwero District in Central Uganda by describing how the CBHI scheme operates, the benefits insured members derive from it and the constraints experienced in retention in the scheme.

## Methods

### Research design

In this paper we report qualitative findings from a larger mixed-methods study seeking to understand the extent of adoption of a community-managed CBHI by community members in a rural district in central Uganda. In this qualitative component we sought to document the nature of the insurance scheme and the experiences of schemes members in participating in the scheme with regard to perceived benefits and barriers to retention in the scheme.

### Study population and sample selection

Study participants comprised of rural community members mainly engaged in informal livelihoods in the central Uganda [19]. Luwero district is made up of a predominantly rural population the majority of whom are dependent on subsistence agriculture as their main source of livelihood. Only about 16% of the households are engaged in formal employment income.

Luwero district was purposively selected because it has one of the oldest operating CBHI union/network of schemes known as *Muno mubulwadde* union of schemes created in 2006. We purposively selected two (of the ten) administrative units that make up Luwero district of which two parishes of Kigombe and Bwaziba under *Munno Mubulwadde* union of schemes purposively selected because they were among the first schemes created between 2005 and 2011 and hence have considerable experiences in community-led and managed CBHI schemes.

We selected members of community groups who had been enrolled in this CBHI scheme for a period of at least five years and had substantial experiences to share regarding the benefits of enrolling in these schemes and the constraints experienced in being a member.

### Data collection methods

#### In-depth interviews

We conducted face-to-face interviews with 18 insured members of *munno mubulwadde* CBHI scheme, in their households, to elicit their experiences of membership of the insurance schemes in terms of benefits and the constraints encountered. The interview guide used in conducting the interviews is attached. In addition, we interviewed 12 members who held leadership positions within the administrative structure of the CBHIs and had intimate knowledge of the day-to-day running and operational aspects of the schemes.

### Focus group discussions

We held four Focus Group Discussions (FDGs) entailing two gender-disaggregated FGDs in each of the two parishes of Luwero district in Central Uganda (one for women and one for men) using a pre-tested focus group guide which is attached [See supplementary file 1]. Each FGD comprised of nine participants from the two parishes of Bwaziba and Kigombe. Respondents for each FGD were purposively selected with the help of community scheme team leaders where, a male/ female team leader helped identify eight participants, four members and four nonmembers of CBHI within his community with him inclusive making a team of nine participants. The FGDs were gender-disaggregated to enable females to express themselves without fear of their male partners. The FGDs were conducted by the first author who has an academic background in the social sciences.

### Data analysis

We followed the procedures recommended for qualitative data analysis by Miles and Huberman (1994). All IDIs and FGDs were audio-recorded and transcribed verbatim into text transcripts. Data analysis was performed through four steps although in actual practise this was an iterative process. First, two authors (CN, HZ) read the transcripts multiple times for data familiarization. Second, the two authors inductively devised a coding scheme from the data and applied it to all the transcripts, using Nvivo 10 software for data management. Third, two authors (CN,HZ) abstracted the inductively coded data into thematic matrices. Disagreements in assignment of codes and themes were resolved through consensus. Fourth, all authors participated in the overall interpretation and synthesis of the results.

## Results

### Demographic characteristics of study participants

In terms of gender, females were more than half of our study participants (52.4%).

With regard to age, the majority of study participants (51%) were in the 30–39 age bracket.

The majority of study participants (84%) had completed a primary school or elementary education while only 8% had attended secondary school or higher.

In terms of marital status, most study participants (82%) were married, 10% were widows and 8% were single.

### Design and structure of *munno mubulwadde* CBHI scheme

*Munno mu Bulwadde* (Your friend in need) Union of Schemes Organization (MBUSO) was established in 2006. MBUSO comprises of 10 CBHI sub-schemes that signed a cooperative agreement for risk pooling across their various memberships in order to achieve economies of scale.

**Fig. 1:**
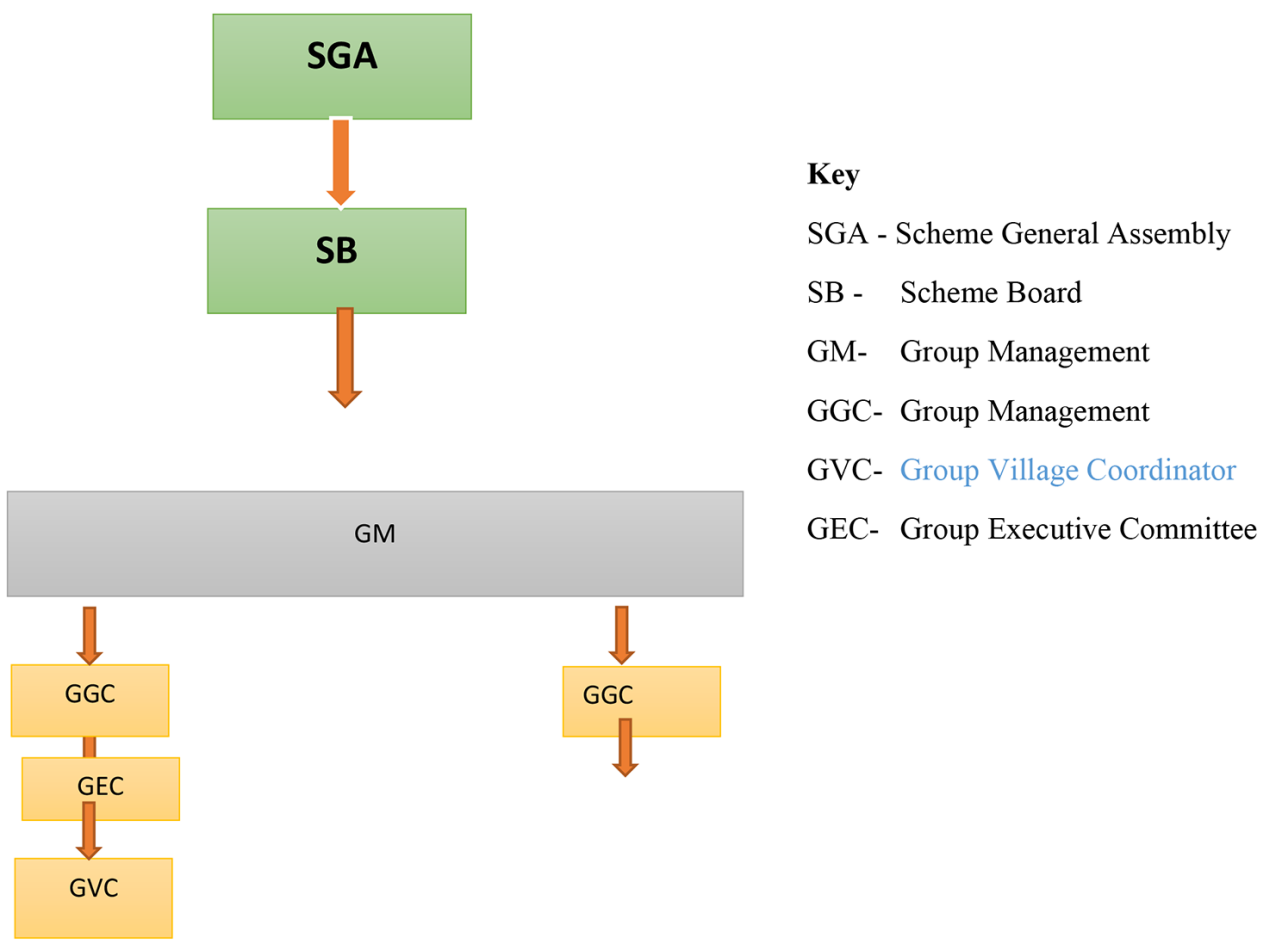
Organogram of MBUSO. Organogram *Munno Mu Bulwadde* Scheme Structure– Luwero District

MBUSO is a voluntary CBHI scheme targeting rural residents in Luwero District in central Uganda. The scheme is formally incorporated in Uganda and has an elaborate organizational structure (Fig. 1).

### Premium payment arrangement

**Fig. 2:**
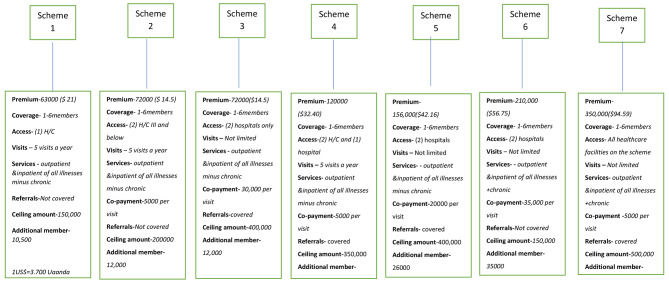
MBUSO insurance packages. The different insurance packages provided under Muno Mubulwadde Union of Schemes in Luwero district

The packages for community members on offer depend on the premium amount as shown in Fig. 2.

The annual premium is payable between November and December every year at designated MBUSO offices although there were plans to change this to July every year to align with maize harvesting season in Uganda. A receipt is issued after which a health access card is issued to the insured member to be presented at the contracted provider. An alternative premium payment mechanism is through separate community saving groups or partner micro-financing institutions operating in the study district. Representatives of MBUSO indicated that they make deliberate efforts to enable their membership afford premiums such as through trainings on enhancing household incomes.

*“To continuously boost household incomes and capacity to pay for health insurance, we initiated relationships with partner micro-financing institutions which still extend micro credit facilities to members of CBHI scheme. We also hold trainings to equip family heads with vocational skills for household livelihood improvement’’* [MBUSO, programme officer, 03].

### Registration procedures

Representatives of MBUSO indicated that those they enroll in membership are households and not individuals. The scheme runs an open recruitment policy for all households in the community willing to pay the premium. Once a member, the family pays an annual health insurance premium which provides medical cover of each of the members in their family. Each household is given identification cards for the registered family members to access health care services from the 13 contracted private providers in Luwero District. Premium covers 1–6 household members cutting across all packages and for any additional member an extra fee is required depending on the package rates. At each of the 13 facilities contracted by the scheme, a special desk has been availed to ease triage processes for scheme members.

Enrollment is done once a year usually during the months of November and December. During enrollment process, new entrants are recruited and renewal of subscription is done for those already on the scheme. Households with less than six members still pay the same premium as those exceeding six family members.

### Co-payments procedures

On top of the insurance premium, the scheme requires members to pay a set fee each time they visit a contracted provider. This fee is called a ‘co-payment’ and the aim is to limit abuse of health insurance through encouraging scheme members to use the nearest public facilities for minor illnesses and reserve major cases for the insurance coverage.

### Perceived benefits of scheme membership

#### Collective or group savings for medical cover

The majority of scheme members we interviewed joined the scheme because of the financial cover provided during episodic illnesses by among members of their household. Participants reported that through the premiums they paid for health cover, they were often not worried about having huge sums of cash to pay such as when their children were down with malaria which was common. In the in-depth interviews, scheme members indicated that their financial contributions through the community health financing initiative introduced by MUBUSO cushioned them from excessive expenditure on medical bills during unexpected bouts of illness or even in event of hospitalization. This they said protected them from slipping into poverty which boosted household financial stability. Members we interviewed also indicated that the community insurance scheme enabled them to make small installment payments for their insurance cover which after some time grew into substantial savings for financial protection during ill-health. One scheme member had the following to say;

*“I had not joined at first because of high premiums, I couldn’t manage paying the money at once, but when group savings were introduced, I was encouraged to join because to me it was like paying premium in installments”* [Male, 32, Scheme member].

#### Perceived better quality health care

Scheme members described receiving better quality health care under the insurance scheme compared to the period they were un-insured. Participants indicated that the insurance cover enabled them to seek better quality health care from private providers. The scheme was said to cover a broad range of health packages.

*“What attracted me was the quality of health care facilities. The scheme contracts some of the best hospitals in our area…we are handled very well at the health care facilities and our packages cover a wide range of illnesses. We even have a client desk specifically for scheme members”* [FGDs, 02, scheme members].

#### Reduced health financial risk

A common refrain from participants was the notion that having health insurance cover provided them with protection against unplanned expenditure during unpredictable episodes of sickness. Considering that these were rural households that largely depended on subsistence agriculture and did not have huge financial reserves, the insurance scheme enabled them to have financial stability during illness by members of their household.

*“The medical services are good and save us from spending too much when we fall sick…recently a neighbor of mine who has just joined the scheme shared with me how having her insurance card saved her from paying a bill of shillings 160,000($44) when she was requested to do a scan and x-ray at the health center”* [Female 28, scheme member].

#### Cover during febrile illnesses

Luwero district in Central Uganda has a high fertility rate and many of the households we visited had mothers who had newborns that they perceived as prone to sickness and hospitalization. Participants indicated that having a baby in a household increased the risk of illness events and hence the need to seek medical care. They mentioned that the insurance scheme was a wise financial decision for any household with a baby due to the frequency of illness that is common with this age group.

Frequent illness of children under five children was cited by households as a major reason for enrolling for the CBHI scheme.

*“I used to spend a lot of money on treatment because my kids frequently fall sick and at times, they could fall sick when I even don’t have a coin…. I struggle as a man and look for money and pay premium knowing I will relax for a full year over medical bills especially for my children”* [Male, 26, scheme member].

### Barriers to retention in scheme membership

#### Long distances to the contracted health care providers

In-depth interviews with administrators of the MBUSO scheme revealed that the long distances between scheme members and the health facilities providing care under the schemes was a fundamental barrier to retention in the scheme by subscribers. As a result of the long distances there was attrition in the scheme membership. In addition, the price fluctuations in the cash crops on which the membership depended such as the falling prices of the maize cash crop meant real incomes were progressively declining. The implication was that falling crop prices meant members were increasingly unable to afford the financial cost of continuing in the health insurance scheme.

*‘’The number of members is reducing due to two factors; ‘the long distances from their homes to the service provider and the decreased incomes due to low prices for maize and coffee crops. This made many drop out’* [Male, 38, Community mobiliser].

#### High cost of premiums

Participants complained about what they perceived as the high cost of premiums which they were increasingly unable to afford due to seasonality and failing crop prices which reduced their real income. They indicated that they struggle to keep up with their financial contribution commitments to the insurance scheme. Seasonality in terms of fluctuations in weather patterns due to dependence on rain-fed crop production was often cited as influential on household revenue which they said was dependent on the vagaries of climate change.

“*We are still facing a lot of challenges first and foremost joining the insurance scheme is voluntary and Luwero being a rural community most people have no stable income and have a lot of other social problems that always seem immediate to attend’* [Male, 33,MBUSO scheme administrator].

#### Untimely premium payment schedule

Schemes members expressed frustration with fixed premium payment schedules which are not well aligned with the harvest seasons of the cash crops they depend on for their livelihoods. Participants suggested the need to make quarterly payments for their payments instead of monthly.

“*They should collect the money during the bean, maize or coffee harvesting seasons not in November or December when we don’t have money’’* [Female, 28, Insured Member MBUSO].

#### Limited information on scheme details

The knowledge community members have about CBHI schemes is very low and scanty. A number of the community members indicated they didn’t have basic knowledge of the scheme and its benefit package and modalities of membership and this was identified as a barrier to wider enrollment into the scheme.

“*The idea of CBHI schemes is not very new here. Our rural community members do not have enough information about how it operates this makes it hard for them to join due to ignorance and others perceive this as an extortion scheme’’* [FGD 04, Scheme members].

## Discussion

We document a novel voluntary community-based health insurance scheme in rural central Uganda involving a union of schemes of insured members. Insured members, the majority of whom belonged to the informal sector, indicated that the benefits of the scheme include reduced out-of-pocket expenditure during episodic events of sickness by household members, financial risk protection cover during frequent and chronic febrile illnesses, perceived better quality care from insurance-contracted providers compared to the nearest public facilities. On the other hand, insured members identified barriers to retention in the insurance scheme such as declining real incomes due to the falling prices of cash crops on which they depend which made it increasingly harder to pay premiums, long distances to scheme-contracted health care providers, untimely premium payment schedules which are misaligned with cash crop harvest seasons and declining affordability of insurance premiums over time and even occasional drop out.

Most studies in Uganda document *provider-based* community-based health insurance schemes such as studies by Agasha et al. [20], Kakama et al. [21] and Twikirize et al. [22] in South Western Uganda. There is relatively less evidence on community-led insurance schemes among low-income earners in rural Uganda. Our study is a contribution to the literature on community-led, community-derived financing and member-managed CBHIS in Uganda which makes a contribution to health financing pathways to advancing the universal health coverage agenda in low-income countries. A study conducted by Kinney and colleagues [23] documents innovations in complimentary financial contributions by inhabitants of islands on Lake Victoria in Southern Uganda in financing HIV treatment and primary health care. However, this scheme is led and managed by a civil society organization.

In this study, participants complained of long distances to insurance-contracted health care providers in rural Luwero district. Our findings mirror those of Kakama and colleagues [21] who report that insured members of a scheme attached to Kisiizi Hospital, a large mission hospital in South-Western Uganda, felt it was too distant from their homes as did insured members in a similar CBHI scheme in Ethiopia [24]. Further still, this study highlights the challenges of declining affordability of insurance premiums by members most of whom depended on agrarian livelihoods. A study in Kenya by Maritimi and colleagues found similar constraints around affordability of health care insurance in Kenya [25].

We found that insured members perceived health services provided by private providers to be of a higher quality than those offered in public facilities in the study district in central Uganda. A study by Twikirize and O’Brien [22] in Uganda found that rural households bypassed public facilities which offered free health services and opted for private facilities where they paid user fees owing to chronic stock out of essential commodities, congestion and demotivated health workers in the public health sector [26]. Studies show that in total, 96% of private health services in Uganda are paid for by households. Hence, out-of-pocket expenditure accounts for one the highest sources of health expenditure (41%) in Uganda [27].

Febrile illnesses emerged as an important motivation for seeking health cover in our sample of participants due to the reported frequencies of outbreaks to households with children in this part of Uganda. The role of frequent childhood illness or morbidity and the economic burden of treatment on households has been raised by several studies [28–30].

Our study adds to the discourse on competing alternatives to health care financing in Uganda in the context of low public finance investments in health care [31], and high out-of-pocket expenditure in Uganda [32, 33]. Uganda’s total budgetary allocation to health has been increasing over the years but spending per capita on health is steadily declining and currently stands at 7% of the overall national budget commitments per year [34]. Our study adds qualitative evidence to the discourse on household struggles on meeting the cost of health care in Uganda. Although the Ugandan legislature passed the National Health Insurance Scheme (NHIS) bill providing for a national insurance scheme in 2019, this has not been assented to by the Ugandan presidency despite a protracted process of multi-stakeholder engagement underpinning its enactment by parliament. Our study offers complimentary approaches to financing universal health coverage in Uganda with relevance to similar settings especially in Sub-Saharan Africa.

In this study participants who depended on the agrarian cash crop economy in central Uganda complained of declining affordability of insurance premiums on account of price volatility of agricultural produce particularly maize and coffee which calls for policy strategies for price stability and other interventions such as the use of farmer cooperative schemes to improve price of this produce [35–39].

Although the MBUSO scheme has been in existence since 2006, the scheme officials indicated that enrollments were still sub-par and that there was a trust deficit from potential members. We recommend interventions aimed at promoting enrollment in community-managed health insurances schemes such as grass root demand creation campaigns, financial incentives for enrollment and the use of social media innovations to increase uptake in the informal sector as well as promoters such as civil society organizations [17].

### Electronic supplementary material

Below is the link to the electronic supplementary material.

**Supplementary Material 1:** Interview guides.

## Data Availability

The datasets generated during and/or analyzed during the current study are not publicly available due to ethical reasons but are available from the corresponding author on reasonable request.

## Abbreviations

CHBI: Community Based Health Insurance
MBUSO: Munno Bulwadde Health Insurance Organization
UHC: Universal Health Coverage
OOP: Out of Pocket Expenditure
SSA: Sub-Saharan Africa
WHO: World Health Organization
