# Supplementary material for: Experiences of membership in munno mubulwadde (your friend indeed) - a novel community-based health insurance scheme in Luwero district in rural central Uganda
Source: BMC Health Serv Res. 2024 Jan 17;24:89. doi: 10.1186/s12913-023-10517-4 (PMC10792776; doi:10.1186/s12913-023-10517-4)
Supplement: Supplementary file 1 — Supplementary Material 1: Interview guides. [file 12913_2023_10517_MOESM1_ESM.docx]

**EXPERIENCES OF MEMBERSHIP IN *munno mubulwadde (your friend indeed)* A NOVEL COMMUNITY-BASED HEALTH INSURANCE SCHEME IN LUWERO DISTRICT IN RURAL CENTRAL UGANDA**

# QUALITATIVE INTERVIEW GUIDES

# Appendix A: Focus Group Discussion Guide- Scheme Members

***Section A***: Level of uptake of community-based health insurance in informal sector in Uganda

Being members of CBHI scheme, how did you get to know about the scheme?

What motivated you to join the scheme?

What benefits have you realized as members of CBHI scheme since you joined?

Would you say most people in your community use CBHI as a means to cover health care costs?

Which are the alternative health financing options to cover health care costs apart from the CBHI?

If yes, what are they?

***Section B***: Challenges in adoption of community-based health insurance in the informal sector

Comment on the design of your CBHI scheme.

what challenges do you face as scheme members?

How do you rate the quality of health care services offered through the community health insurance schemes?

What is your opinion about amount of premium paid in the scheme?

What are the barriers to uptake of community-based health insurance?

***Section C***: Strategies for promoting the adoption of community-based health insurance in the informal sector

How best can health insurance knowledge be passed on?

What favourable fee needs to be charged on the health insurance scheme per month?

How would you prefer to pay subscription fees for health insurance?

What do you think can be done to encourage other community members to join the scheme?

# Appendix B: Focus Group Discussion Guide- Non-Scheme Members

***Section A***: Level of uptake of community-based health insurance in informal sector in Uganda

A .1 Do you know of any community-based health insurance scheme in your community?

A .2 How many have ever been members of any community-based health insurance scheme and now no longer members?

A .2.1 If any, why did you drop out of the schemes?

A .3 Would you say most people in your community use CBHI as a means to cover health care costs?

A .4 Describe by character the kind of families that have enrolled for CBHI in this community?

***Section B***: Challenges in adoption of community-based health insurance in the informal sector

B .1What makes it difficult for you to join community-based health insurance scheme?

B .2What is your opinion about amount of premium paid in the scheme?

B .3Rate the relevance of Community Based Health insurance scheme to your community

Section C: Strategies for promoting the adoption of community-based health insurance in the informal sector

C .1What are the means of covering health care costs in your families?

C .2How best can health insurance knowledge be passed on?

C .3 What do you think can be done to encourage community members to join the scheme?

# Appendix C: Interview Guide I: Administrators of the CBHI Coordinating Institution (Muno Mu Bulwadde Union of Schemes)

A. How do you rate the uptake of CBHI in Luwero district?

A i If response in 1a is high, what indicates the high uptake rates/ success of CBHI?

A ii. If response in 1a is average/ low, what do you think are the possible causes for the average/ low up take of CBHI?

B. Who qualifies to be a member in a community-based health insurance scheme?

C. Comment on the cost of health insurance scheme

D. What achievements have you realized as a union of CBHI shames since inception?

E. Describe the management and administrative structure of schemes under this union

F. Please give a back ground of Muno Mubulwadde union of schemes

G. As a union what key constraints have you encountered during the process of promoting CBHI in Luwero district

Institutional level

Scheme level

Service provider level

Community levels

H. What do you think can be done to improve adoption of CBHI in Luwero district?

# Appendix D: Interview Guide II: CBHI Scheme Service Provider (Hospital)

A. What is the estimated population served in the CBHI scheme?

B. To what extent has CBHI schemes impacted on access of health care in Luwero District?

C. Give reasons for your answer in 3 above

D. Please comment on the health care package provided to CBHI scheme beneficiaries in this hospital

E. What challenges is the hospital facing as a result of working with CBHI scheme(s)

F. What are the common complaints by scheme members about service provided at this health Centre the impact on their interest to seek health services?

G. Comment on the impact of the partnership between this hospital and the insurance scheme on health care service delivery

Hospital administration

Scheme administration

H. What do you think can be done to improve on the uptake of community health insurance?

# Appendix E: Interview Guide III: CBHI Scheme Coordinating/ Mobilizing Staff at Community Level

A. How would you tell that your scheme is effective?

B. What factors affect the level of uptake of CBHI in this community?

C. For how long have you served as a leader in this scheme?

D. Please describe the structure of your scheme design

E. Who qualifies to be a member in a community-based health insurance scheme?

F. Are members of this scheme involved in the management of the insurance scheme?

G. Are people willing to pay health insurance in informal sector outside their group of friends and relatives?

H. What is the saving culture of people in this community (what is the Socio-economic profile of households/beneficiaries of this scheme in your community?

I. Comment on the quality of information provided to people in informal sector about health insurance

J. What has failed the effectiveness of community-based health insurance?

K. What can be done by the government to improve community-based health insurance?

L. What can be done by policy makers to improve Non-formal health insurance?
